# Smoke-Free Policies at Home, Church, and Work: Smoking Levels and Recent Quit Attempts Among a Southeastern Rural Population, 2007

**Published:** 2011-12-15

**Authors:** Carla J. Berg, Deanne W. Swan, Michelle C. Kegler, George Fredrick, Sandra Daniel

**Affiliations:** Department of Behavioral Sciences and Health Education, Emory University; Emory University, Atlanta, Georgia; Emory University, Atlanta, Georgia; Phoebe Putney Memorial Hospital, Albany, Georgia; Georgia Southwestern State University, Americus, Georgia

## Abstract

**Introduction:**

The objective of this study was to examine the cumulative effect of smoke-free policies and social support for smoking cessation in the home, at church, and at work on smoking levels and quit attempts in the context of a community-based study of rural African Americans and whites in the Southeast.

**Methods:**

We conducted a baseline survey to assess sociodemographics, smoking behavior, level of social support for smoking cessation, and smoke-free policies at home, church, and work. We created a variable for a weighted "dose" of smoking restrictions on the basis of the existence of policies in the 3 settings and a weighted score for social support and used bivariate analyses and multivariate regression to analyze data.

**Results:**

Of 134 survey participants, 18.7% had complete restrictions at home. Among church attendees, 39.4% had complete restrictions at church, and among those employed outside the home, 15.4% had complete restrictions at work. After controlling for age, sex, race, and education, the weighted dose of smoking restrictions was significantly related to having made a quit attempt in the past 12 months (odds ratio, 2.2; 95% confidence interval, 1.1-4.3) but not number of cigarettes smoked per day. Social support for cessation had no effect on recent quit attempts or number of cigarettes smoked per day.

**Conclusion:**

Smoke-free policies have a cumulative effect on smoking behavior. These findings may inform interventions aimed at promoting comprehensive community-wide smoke-free policies.

## Introduction

Cigarette smoking remains one of the leading causes of preventable disease in the United States (1). Since the 1986 Surgeon General's report, government agencies have established policies restricting smoking in public areas (eg, churches, workplaces) (2). By 1999, 69% of US workers had smoke-free workplaces (3). Having workplace smoking restrictions is associated with a lower prevalence of smoking, higher quit rates, more recent quit attempts, and lower cigarette consumption (4), and complete restrictions have more effect than partial restrictions (5). Research on the effects of faith-based organizations implementing restrictions on smoking is lacking, despite the important social role such organizations play (6), particularly in rural areas, where 60% of US congregations are located (7). Qualitative research has documented social influences of church leadership and friends on physical activity and healthy eating in church settings (8).

Despite the health benefits of public smoke-free policies (9), the prevalence of these policies varies. Certain geographic areas, such as the Southeast (typically the tobacco-growing states), are less likely to have stringent workplace policies, which likely also vary by occupation, industry (10), and employee socioeconomic status (11). For example, blue-collar workers may have higher levels of exposure to environmental tobacco smoke than other kinds of workers because smoking restrictions are often not strictly enforced in blue-collar settings (12). Thus, examining the existence of smoke-free public policies in different settings is important.

The prevalence of home smoking restrictions has also increased (13) but still varies widely (14). For example, people in rural counties are more likely to smoke, have fewer smoking restrictions at home, and are more willing to accept exposure to environmental tobacco smoke than people in urban counties (15-17). Moreover, racial/ethnic minority populations, particularly African Americans, and people with lower socioeconomic status may also be less likely to have smoke-free homes (18).

Having smoke-free policies in the home is associated with reduced exposure to environmental tobacco smoke, fewer occurrences of smoking in the household (19), lower levels of smoking, and lower concentrations of air nicotine and urinary cotinine (20). Having a smoke-free home is also related to fewer cigarettes smoked per day (21,22) and less nicotine dependence (23). Household restrictions have been positively associated with more prior quit attempts (18,22), interest in quitting (18), long-term abstinence, and relapse prevention (19).

Another factor associated with smoking and quitting behavior is social support (24). Successful quitters have higher levels of support from partners than smokers who relapse or never quit (24,25). The prevalence of smoking is higher among separated or divorced people than among people currently married or widowed (26).

Limited research has focused on sources of smoke-free policies and social support for cessation and the cumulative effect of these factors on smoking level and attempts to quit smoking. A social ecology approach predicts that people exposed to a larger number of settings that restrict smoking (indicating a lack of social acceptance to smoking and secondhand smoke exposure) and to more social pressure to quit would have more quit attempts and smoke fewer cigarettes (27). The objective of this study was to examine the relationship between smoke-free policies and social support for smoking cessation in 3 settings (home, church, and work) and smoking behavior (number of cigarettes smoked per day and having made at least 1 quit attempt in the past year) in the context of a community-based study of rural African Americans and whites in the Southeast.

## Methods

Data for this analysis came from the Healthy Rural Communities 2 (HRC2) study (28), a community-based participatory research study conducted by the Emory Prevention Research Center in collaboration with the Southwest Georgia Cancer Coalition. HRC2 was a cross-sectional study that included a baseline survey of 527 participants. Results reported here are from the baseline survey data of current smokers only (n = 134). The study protocol was approved by the Emory University institutional review board. Details on HRC2, including recruitment methods, are provided elsewhere (28).

### Study participants and study setting

Inclusion criteria for participation in the parent study included being African American or white, being aged 40 through 70 years, living with at least 1 other person, and residing in 1 of 4 rural southwest Georgia counties (Brooks, Sumter, Worth, and Decatur) for 5 or more years. Each of the 4 counties has a population of less than 35,000 and a large percentage (roughly 40%) of African American residents (29). The study purposively recruited equal proportions of African Americans and whites and men and women in each county. The study excluded people with a cancer diagnosis within the previous 2 years and non-English speakers.

In 2005, Georgia implemented the Smoke-Free Air Act, which banned smoking statewide in all enclosed workplaces. The Act exempted designated smoking areas in nonwork areas of businesses, bars, and restaurants where people aged 18 years or younger are not employed or permitted entrance; private residences not used as health care or child day care facilities; retail tobacco stores; nursing homes; and privately owned meeting and assembly rooms during private functions where people aged 18 or younger are not permitted. Local governments, however, are allowed to regulate smoking more strictly, and level of enforcement may vary among communities.

### Data collection

Recruitment of HRC2 participants took place at 70 community sites from September 2006 through March 2007. Local research staff approached potential participants in person to ask if they would be willing to take part in a study on how family, church, and work affect health and wellness. Recruitment sites were located in small towns in the 4 study counties and included businesses (n = 11), restaurants (n = 8), churches (n = 7), libraries (n = 5), police and sheriff's offices (n = 4), social service agencies (n = 4), other government agencies (n = 8), civic organizations (n = 3), county health departments (n = 2), multiunit housing complexes (n = 2), and city halls (n = 2). Additional sites included a technical college, a YMCA, a community college, a health clinic, a park, a courthouse, and a county prison. We selected sites on the basis of the range of socioeconomic status among the population and the ability to meet the study's sex and race/ethnicity recruitment objectives. Research staff screened potential participants for eligibility and obtained written informed consent on-site. Participants completed self-administered surveys (also on-site) and were given a $20 gift card as compensation for their time. Approximately 89% of those eligible completed the survey.

### Measures

**Current smokers.** The baseline survey asked, "Do you now smoke cigarettes every day, some days, or not at all?" We defined current smokers as people who reported smoking on some days or every day.

**Sociodemographics, church attendance, and employment status.** We used standard items to assess sex, race, age, marital status, number of people in the household, educational attainment, annual household income, employment status, and church attendance. We asked, "How often do you attend religious services?" Possible responses were "never or almost never," "a few times a year," "a few times a month," and "at least once a week." We categorized this variable as "attended church regularly" if they reported attending at least a few times a month. We asked participants, "What is your employment status?" Possible responses were "employed part-time," "employed full-time," and "not employed." We asked, "Do you work outside the home?" Possible responses were yes and no. We considered participants to be working outside of the home if they reported being employed full-time or part-time and working outside of the home.

**Predictor variables.** Our 2 main predictors were smoke-free policies and social support for cessation in the 3 settings of home, work, and church. To assess smoke-free home policies, we asked participants, "Which statement best describes the rules about smoking inside your home?" Possible responses were "smoking is not allowed anywhere inside your home," "smoking is allowed some places or at some times," "smoking is allowed anywhere inside your home," and "there are no rules about smoking inside your home" (30). To assess smoke-free policies at work and at church, we asked, "Does your church/worksite have a smoking policy or rule that says . . . ?" Possible responses were "smoking is not allowed anywhere," "smoking is allowed only in a few smoking areas," "smoking is allowed anywhere except a few nonsmoking areas," and "there is no policy/rule" (30). We defined complete restrictions as "Smoking is not allowed anywhere." We created a variable for weighted "dose" of smoke-free restrictions for each study participant. For each setting (home, church, work) to which the participant was exposed, we assigned a numeric score to designate a level of smoking restriction (0 = no restrictions, 1 = partial restrictions, and 2 = complete restrictions). We added the score for each setting to calculate a total dose (possible scores ranged from 0 to 6) and then divided the total dose by the number of settings to calculate a weighted dose. For example, a participant who has complete restrictions at work and at church but only partial restrictions at home would have a total dose of 5 and a weighted dose of 1.7 (5 divided by 3 settings). The weighted dose of smoking restrictions ranged from 0 to 2.

To assess social support for cessation, we adapted the Partner Interaction Questionnaire (31). We selected items most likely to reflect common occurrences in each setting; we altered wording slightly for the church and work settings. To assess the home setting, we asked, "During the past 6 months, how often did your family or anyone living in your household do or say the following?" Possibilities were, "asked you to quit smoking," "commented on your lack of willpower," "refused to let you smoke in the house," "mentioned being bothered by smoke," and "criticized your smoking." To assess the church setting, we asked, "During the past 6 months, how often did anyone from your church do or say the following?" Possibilities were, "asked you to quit smoking," "commented that smoking is a dirty habit," and "criticized your smoking." To assess the work setting, we asked, "During the past 6 months, how often did your coworkers say or do the following?" Possibilities were "asked you to quit smoking," "commented that smoking is a dirty habit," and "criticized your smoking." For each item, participants responded according to a 4-point Likert scale (0 = never or rarely, 1 = sometimes, 2 = often, or 3 = almost always). For each subscale (home, church, work), we averaged the items to create 1 composite score for social support for smoking cessation with a range of 0 to 3, with higher scores indicating higher levels of social support. The Cronbach α for the home subscale was 0.82; for the church subscale, 0.88; and for the work subscale, 0.88. We created a weighted score of social support by totaling the scores for each subscale (home, church, and work) and dividing by the number of settings to which a participant was exposed. For a participant who did not work outside of the home, the social support scores for home and church were added together and then divided by 2. For example, a participant who has a social support score of 2 at home and a score of 3 at church would have a weighted score of 2.5. The weighted score for social support ranged from 0 to 3.

**Outcomes.** There were 2 outcomes: number of cigarettes smoked per day and having made at least 1 quit attempt in the past year. We asked participants*,* "During the last 12 months, have you stopped smoking for 1 day or longer because you were trying to quit smoking?" (30) and "On average, how many cigarettes do you now smoke per day?" (30).

### Statistical analyses

We summarized participant characteristics using means and standard deviations for continuous variables and numbers and percentages for categorical variables. We conducted bivariate analyses to examine whether quit attempts or cigarettes smoked per day varied by sociodemographic characteristics. We used binary logistic regression to model the effect of smoking restrictions and social support on quit attempts and ordinary least squares regression to model the effect of smoking restrictions and social support on number of cigarettes smoked per day. In both models, we controlled for age, sex, race, and education. We entered our primary predictors of interest — smoke-free policies and social support for cessation, weighted across settings — into the model after controlling for sociodemographics. We excluded from analysis people who were missing data on outcomes (listwise deletion). To retain as many participants as possible in the analysis, other missing data, including sociodemographics and predictors, were imputed using a hot-decking algorithm in SAS/IML. We conducted all analyses with SAS version 9.2 (SAS Institute Inc, Cary, North Carolina).

## Results

Among the 134 participants, 78.2% attended church regularly, 68.4% worked outside of the home, and 18.7% had complete restrictions at home. Among regular church attendees, 39.4% had complete restrictions at church, and among participants employed outside the home, 15.4% had complete restrictions at work (Table 1).

Attempting to quit in the past year was related to being African American, being younger, having a lower score for social support, and having a greater dose of smoking restrictions (Table 2). Smoking more cigarettes per day was related to being white and a having a lower dose of smoking restrictions. The multivariate regression model fit the data (*F*_6,122_ = 7.32; *P* < .001) and explained 26.5% of the variance in number of cigarettes smoked per day (Table 3). After controlling for age, sex, race, and education, the weighted dose of smoking restrictions was significantly related to having made a quit attempt in the past 12 months (odds ratio, 2.2; 95% confidence interval, 1.1-4.3) but not number of cigarettes smoked per day.

## Discussion

No previous research has examined the cumulative effect of the various settings for smoke-free policies and social support on individual smoking level (ie, number of cigarettes smoked per day) and recent quit attempts. We found that the overall weighted dose of smoke-free restrictions at home, at church, and at work were significant predictors of quit attempts in the past year, but not of smoking level, after controlling for other important sociodemographic characteristics. Social support for cessation in these settings was not significantly related to these outcomes.

Our study is one of the first to document the prevalence of smoke-free policies in churches, and it is notable that churches most commonly had complete restrictions. The prevalence of complete restrictions at home in our sample of rural smokers (18.7%) is lower than reported in other research: 25.4% among a sample of rural smokers in Kansas (21) and 22.0% among rural, low-income smokers in Oklahoma (22). Likewise, the prevalence in our sample of complete restrictions at work (15.4%) is much lower than a national rate (69%) reported in 2001 (3). The lower rate in our sample is alarming because our data should reflect the increase in smoke-free policies implemented in the past 10 years.

We found a cumulative effect of restrictions in the 3 settings on recent quit attempts but not smoking level. While our findings on the relationship between smoking restrictions and quit attempts is in line with previous research, the lack of a relationship between smoking restrictions and smoking level contradicts previous research. For example, smoking restrictions at work have been related to a decrease in smoking level and more numerous and successful quit attempts (4). Home restrictions have been related to fewer cigarettes smoked per day (22), more prior quit attempts (22), interest in quitting (18), long-term abstinence, and relapse prevention (19). Both home policy and public policy (ie, at church and at work) play a cumulative role in decreasing not only levels of environmental tobacco smoke but also the health risks of smoking.

Interestingly, social support for quitting was not significantly associated with smoking level or quit attempts. Research has consistently documented the correlation between social support and success in smoking cessation (32). However, research has also documented that social support for cessation from a smoker is less effective than support from a nonsmoker (33). Our study did not assess the smoking behavior of people providing support; perhaps our study participants received support from smokers.

This study has implications for research and practice. First, the cumulative effect of smoke-free policies in the various settings argues for comprehensive health campaigns involving the promotion of smoke-free policies in multiple contexts. Second, further examination is warranted on the role of social support in different settings to determine the kinds of social support that are most effective and whether social support is more effective from one group of people than from another group. Finally, further research should use a large sample to determine the effect of each setting on smoking behavior.

This study has limitations. First, our data are cross-sectional, thus limiting our ability to infer causality. Second, our measures were self-reported, and self-report can introduce social desirability bias. Third, our sample size did not allow testing of all of the relationships in the multivariate model that a larger sample size could have addressed. Finally, this study represents a convenience sample. Study participants were possibly more health conscious than others living in the same rural communities or different in some other respect.

We found a cumulative effect of smoke-free policies on attempts to quit smoking but not smoking level among our rural sample, despite the fact that social support was not significantly associated with these outcomes. Thus, health promotion interventions focused on reducing the prevalence of smoking and decreasing amounts of exposure to environmental tobacco smoke may benefit from promoting smoke-free policies in multiple settings, including homes, churches, and workplaces.

## Figures and Tables

**Table 1:** Characteristics of Self-Reported Current Smokers in Healthy Rural Communities 2 Study at Baseline (N = 134), Southwest Georgia, 2007^a^

**Characteristic**	No. (%)
**Sex**	
Male	76 (56.7)
Female	58 (43.3)
**Race**	
Non-Hispanic white	66 (49.3)
Non-Hispanic African American	68 (50.7)
**Marital status**	
Married or living with partner	67 (50.0)
Other	66 (49.6)
**Education**	
Less than 12th grade	22 (16.4)
High school graduate or General Educational Development	50 (37.3)
Some college or technical school	41 (30.6)
College graduate	21 (15.7)
**Annual household income, $**	
<10,000	20 (14.9)
10,000-24,999	37 (27.7)
25,000-49,999	26 (19.4)
≥50,000	25 (18.7)
Missing data	26 (19.4)
**Attends church regularly**	104 (78.2)
**Works outside the home**	91 (68.4)
**Complete smoking restrictions**	
Among all survey participants	
At home	25 (18.7)
At church	41 (30.6)
At work	14 (10.4)
Among subsamples	
Attend church regularly (n = 104)	41 (39.4)
Employed outside the home (n = 91)	14 (15.4)

**Characteristic**	**Mean (SD)**

**Age, y**	49.0 (7.2)
**Social support for cessation, score^b^**	
At home	1.7 (0.9)
At church	2.4 (0.9)
At work	2.4 (0.8)
**Social support, weighted score^c^**	2.1 (0.7)
**Smoking restrictions, weighted dose^d^**	1.1 (0.6)

Abbreviations: SD, standard deviation.

a Not all categories add to 134 participants because of missing data.

b Ranged from 0 to 3, with higher scores indicating greater social support.

c Ranged from 0 to 3, with higher scores indicating greater social support.

d Ranged from 0 to 2, with higher scores indicating a greater dose.

**Table 2:** Recent Quit Attempts and Number of Cigarettes Smoked per Day, by Sociodemographic Characteristics and Predictors Among 134 Current Smokers in Healthy Rural Communities 2 Study at Baseline, Southwest Georgia, 2007^a^

**Sociodemographic Characteristic**	Quit Attempt in Past Year			Cigarettes Per Day	

	Yes, No. (%)	No, No. (%)	*P* b	Mean (SD)	*P*
**Total**	64 (47.8)	70 (52.2)	NA	16.0 (12.2)	NA
**Sex**					
Male	35 (26.1)	41 (30.6)	.65	16.8 (10.4)	.35^c^
Female	29 (21.6)	29 (21.6)		14.9 (13.5)	
**Race**					
Non-Hispanic white	23 (17.2)	43 (32.1)	.003	21.2 (11.3)	<.001^c^
Non-Hispanic African American	41 (30.6)	27 (20.1)		10.9 (11.0)	
**Age, y**					
40-49	44 (32.8)	34 (25.4)	.01	14.6 (10.7)	.09^d^
50-59	12 (9.0)	30 (22.4)		19.3 (15.0)	
60-70	8 (6.0)	6 (4.5)		13.2 (8.4)	
**No. in household**					
2	16 (11.9)	19 (14.2)	.37	18.0 (13.0)	.05^d^
3	18 (13.4)	24 (17.9)		15.4 (12.6)	
4	12 (9.0)	16 (11.9)		19.7 (13.3)	
≥5	18 (13.4)	11 (8.2)		11.2 (8.0)	
**Marital status**					
Never married	2 (1.5)	4 (3.0)	.14	12.7 (11.0)	.05^d^
Married/living with partner	27 (20.3)	40 (30.1)		18.7 (12.9)	
Other	34 (25.6)	26 (19.5)		13.6 (11.0)	
**Education**					
Less than 12th grade	11 (8.2)	11 (8.2)	.10	11.9 (10.0)	.31^d^
High school graduate or General Educational Development	28 (20.9)	22 (16.4)		15.7 (12.9)	
Some college or technical school	20 (14.9)	21 (15.7)		17.9 (10.6)	
College graduate	5 (3.7)	16 (11.9)		17.1 (14.9)	

**Predictors**	**Mean (SD)**	**Mean (SD)**	***P* c**	***r***	***P* e**

Age	48.3 (7.5)	49.7 (6.9)	.24	0.068	.33
Social support, weighted score^f^	1.9 (0.9)	2.3 (0.5)	.002	0.167	.06
Smoking restrictions, weighted dose^g^	1.3 (0.6)	1.0 (0.6)	.002	−0.275	.001

Abbreviations: SD, standard deviation; NA, not applicable.

a Not all categories add to 134 participants because of missing data.

b *P* value determined by χ^2 ^test.

c *P* value determined by independent-samples *t* test.

d *P* value determined by analysis of variance test.

e *P* value determined by *t* test for correlations.

f Ranged from 0 to 3, with higher scores indicating greater social support.

g Ranged from 0 to 2, with higher scores indicating a greater dose.

**Table 3:** Factors in Having Made a Quit Attempt in the Past Year and Cigarettes Smoked per Day Among Smokers in the Healthy Rural Communities 2 Study, 2007

**Variable**	Quit Attempt^a^		Cigarettes Smoked Per Day^b^		

	OR (95% CI)	*P*	*b* (SE)	β	*P*
Age	1.0 (0.9-1.0)	.16	0.11(0.13)	0.06	.42
Sex (referent is male)	1.7 (0.8-3.7)	.21	0.1 (0.1)	0.09	.26
Race (referent is white*)*	2.0 (0.9-4.4)	.10	−3.8 (2.0)	−0.16	.05
Education (referent is high school graduate or less)	0.6 (0.3-1.4)	.28	−9.7 (2.1)	−0.41	<.001
Social support, weighted score^c^	0.6 (0.3-1.0)	.07	1.3 (2.1)	0.05	.53
Smoking restrictions, weighted dose^d^	2.2 (1.1-4.3)	.02	−0.4 (1.4)	−0.02	.80

Abbreviations: CI, confidence interval; OR, odds ratio, SE, standard error.

a Tests of overall fit: likelihood ratio = 23.99, *P* < .001; Wald test = 18.83, *P* = .004; Hosmer-Lemeshow goodness-of-fit test, χ^2^ = 6.87, *P* = .55.

b For full model, *R*² = 0.265, *F*_6,122_ = 7.32, *P* < .001; Δ*R*² = 0.06.

c Ranged from 0 to 3, with higher scores indicating greater social support.

d Ranged from 0 to 2, with higher scores indicating a greater dose.
